# Amyloid Precursor Protein: A Regulatory Hub in Alzheimer's Disease

**DOI:** 10.14336/AD.2023.0308

**Published:** 2024-02-01

**Authors:** Jiang Chen, Jun-Sheng Chen, Song Li, Fengning Zhang, Jie Deng, Ling-Hui Zeng, Jun Tan

**Affiliations:** ^1^Key Laboratory of Endemic and Ethnic Diseases, Laboratory of Molecular Biology, Ministry of Education, Guizhou Medical University, Guiyang, Guizhou, China.; ^2^Key Laboratory of Endemic and Ethnic Diseases, Ministry of Education, Guizhou Medical University, Guiyang, Guizhou, China.; ^3^The First Affiliated Hospital of Dalian Medical University, Dalian, Liaoning, China.; ^4^Key Laboratory of Novel Targets and Drug Study for Neural Repair of Zhejiang Province, School of Medicine, Zhejiang University City College, Hangzhou, Zhejiang, China

**Keywords:** amyloid precursor protein, Alzheimer’s disease;, amyloid-β, secretase, endocytosis, tau protein

## Abstract

Decades of research have demonstrated an incontrovertible role of amyloid-β (Aβ) in the etiology of Alzheimer's disease (AD). However, the overemphasis on the pathological impacts of Aβ may obscure the role of its metabolic precursor, amyloid precursor protein (APP), as a significant hub in the occurrence and progression of AD. The complicated enzymatic processing, ubiquitous receptor-like properties, and abundant expression of APP in the brain, as well as its close links with systemic metabolism, mitochondrial function and neuroinflammation, imply that APP plays multifaceted roles in AD. In this review, we briefly describe the evolutionarily conserved biological characteristics of APP, including its structure, functions and enzymatic processing. We also discuss the possible involvement of APP and its enzymatic metabolites in AD, both detrimental and beneficial. Finally, we describe pharmacological agents or genetic approaches with the capability to reduce APP expression or inhibit its cellular internalization, which can ameliorate multiple aspects of AD pathologies and halt disease progression. These approaches provide a basis for further drug development to combat this terrible disease.

## 1. Introduction

Alzheimer’s disease (AD) is a prevalent form of dementia that affects nearly 50 million individuals worldwide, particularly among the elderly population. This progressive disease causes significant health, economic, and social issues [1]. Amyloid precursor protein (APP) plays an important role in neuronal development, synapse formation, and repair, while amyloid-β (Aβ), a proteolytic metabolite of APP, is involved in synapse formation and plasticity under normal physiological conditions [2]. Both APP and Aβ have been implicated in AD. For example, pathogenic mutations in the APP gene have been found in familial AD (FAD) patients [3]. In sporadic AD (SAD) brain samples, Aβ, a small hydrophobic peptide, is derived from the precursor protein APP [4]. Mutations in presenilin 1 and presenilin 2 genes, which affect the processing of APP and Aβ production, are also common in FAD patients [3]. Dysregulation of APP expression has been described in AD patients, and Down syndrome (DS) is associated with AD-like dementia due to APP triplication [5-7]. However, anti-AD drug development has largely focused on Aβ removal as an anti-amyloid therapeutic approach, despite the failure of almost all phase II/III clinical trials of anti-amyloid agents due to either lack of efficacy or unforeseen toxic side effects [8, 9]. It is also evident that amyloid deposition does not correlate with disease progression [10]. Furthermore, Aβ toxicity has been reported to depend on APP expression, indicating a role for APP beyond the generation of toxic fragments [11-13]. Therefore, many have argued that Aβ is not the sole cause of the disease and that interplay between APP and its fragments is required for the observed toxicities of these molecules [14]. Thus, an important question is whether the loss of APP-mediated physiological functions or the gain of APP-participated pathological functions may contribute to AD symptoms and possibly also to AD pathogenesis [11, 15]. According to the “amyloid cascade hypothesis,” accumulation of neurotoxic Aβ contributes to AD pathogenesis [16]. APP mutations directly affect the production of Aβ, and increased expression or excessive endocytosis of APP protein accelerates the pathological process of AD [17]. Thus, APP plays a direct role in AD development and progression as a crucial regulatory protein. In this review, we discuss the multifaceted role of APP and its metabolites in AD pathogenesis and progression, and advocate for the development of APP-modulating therapies as alternative treatment strategies. We further review the drugs and drug-like compounds already identified to modulate APP.

**Figure 1.**
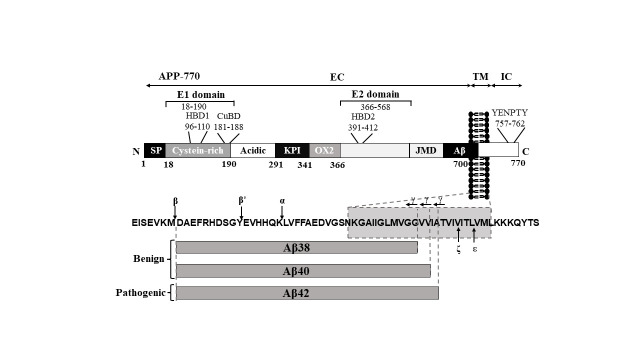
**Structure of APP: APP770 structure and Aβ peptide fragment**. Protein structure (APP770). APP is composed of three domains: extracellular domain (EC), transmembrane domain (TM), and intracellular domain (IC). APP has an N-terminal signal peptide (SP); E1 domain with a heparin-binding domain (HBD1), a copper-binding domain (CuBD); acidic region; APP751 and APP770 contain a Kunitz protease inhibitor (KPI) domain and an Ox-2 antigen domain; E2 domain with a second heparin-binding domain (HBD2). BACE cleaves APP after Met_671_(β) and Tyr_681_(β′), whereas ADAM10 processes APP inside the Aβ peptide sequence after Lys_687_(α). γ-Secretase cleavage in the transmembrane region (TM) yields primarily 38, 40 and 42 amino acid residue-long Aβ peptides Aβ_38_, Aβ_40_ and Aβ_42_. ζ-site Aβ_46_ and ε-site Aβ_49_ downstream of the γ-site proximal to the membrane-intracellular boundary (for details concerning the γ-, ζ- and ε-sites).

## 2. Structure and functions of APP

APP is a type I transmembrane glycoprotein with a large extracellular domain and a short cytoplasmic tail (Fig. 1). The extracellular domain of APP is composed of several subdomains with distinct structural features (Supplementary Table 1). The copper-binding domain (CuBD), which binds copper ions, is composed of a small β-sheet and an α-helix. The heparin-binding domain (HBD), which interacts with heparin and other glycosaminoglycans, is composed of a β-sheet and a flexible loop. The growth factor-like domain, which has structural similarity to some growth factors, is composed of a β-sheet and a short α-helix. The different subdomains of the extracellular domain are arranged in a compact and globular manner, allowing them to interact with various ligands and extracellular matrix proteins. The transmembrane domain of APP is a single-pass α-helix that spans the cell membrane. The helix is amphipathic, with hydrophobic residues facing the membrane and hydrophilic residues facing the cytoplasm and extracellular environment. The transmembrane domain is important for the stability and localization of APP within the cell membrane and is also involved in the interaction of APP with other transmembrane proteins. The juxtamembrane domain within APP, located adjacent to the transmembrane domain. contains a GxxxG motif that promotes the dimerization of APP and interaction with other transmembrane proteins. The cytoplasmic domain of APP contains several conserved motifs, including YENPTY, which interacts with intracellular signaling molecules. The cytoplasmic domain is also involved in the regulation of APP trafficking and processing, and mutations in this domain have been associated with an increased risk of developing AD. Overall, the three-dimensional structure of the different domains within APP plays a critical role in its function and processing and is important for understanding the pathogenesis of AD and other neurological disorders. However, further studies are still needed to fully elucidate the exact structure–function relationships of APP and its interactions with other proteins and ligands.

**Table 1:** Schematic representation of APP structure and function.

APP structural regions	Location	Function	Primary papers
**E1**	exons 1-5: amino acid residues 18-189 (GFLD; CuBD)	hydroxyl radical production, APP dimerization and synaptic adhesion	[24-26]
**AcD**	exons 6: amino acid residues 191-291	unfolded and highly flexible, and directly links E1 to E2	[26]
**KPI**	exons 7: amino acid residues 292-341	be relevant to metabolic enzymes, mitochondrial function and cell growth	[20-23]
**OX-2**	exons 7-8: amino acid residues 344-365	cell-surface binding and recognition	[20]
**E2**	exons 9-14: amino acid residues 374-565 (HBD; RERMS; )	trophic functions	[31-35]
**JMD**	exons 14-17: amino acid residues 366-700	Contains the cleavage sites for α-secretase and β-secretase	[36]
**Aβ**	exons 16-17: amino acid residues 671-712	Regulates neuronal homeostasis; abnormally aggregated forms of Aβ oligomers and plaques;	[41-43]
**TMD**	exons 17: amino acid residues 701-723	γ-secretase cuts; Directly interacts with cholesterol; modulates APP processing;	[39,40]
**AICD**	exons 17-18: amino acid residues 724-770	transcriptional regulator;leads to hippocampal degeneration, tau phosphorylation and deficits in working memory	[44-51]

AcD: acidic domain; KPI: Kunitz protease inhibitor; OX-2: Ox-2 antigen domain; JMD: juxtamembrane domain; TMD: transmembrane domain; AICD: APP intracellular domain; GFLD: growth factor-like domain; CuBD: copper-/metal-binding domain; HBD: heparin-binding domain; RERMS (amino acids 328-332) was uniquely required for the growth-promoting activity of sAPP-695.

APP is encoded by a single gene, and three major isoforms resulting from alternative splicing have been characterized: APP695, APP751, and APP770. The number of amino acids in each isoform determines its isoform [18]. Mammalian APP and its associated proteins have a high degree of similarity with conserved areas, such as the extracellular acidic domain (AcD), E1 and E2 domains and intracellular domain (Table 1) [19]. Unlike other APP homologs, human APP contains the unique Aβ sequence, which is not found in amyloid precursor-like protein 1 (APLP1) or APLP2 [19]. Therefore, APP may be closely related to the maintenance of complex cognitive behavior in the human brain. In contrast to APP751, which lacks the Ox-2 domain, APP695 is devoid of both the Kunitz proteinase inhibitor (KPI) and Ox-2 domains [20]. The Ox-2 domain in APP is thought to be involved in cell-surface binding and recognition [20], while the KPI domain, found in some APP isoforms, is upregulated in the AD brain and has been shown to play a role in the production of Aβ peptides and regulation of blood clotting serine proteases in platelets [21]. Meanwhile, Ox-2 antigen is a glycoprotein on the surface of lymphoid and neural cells that shares similarity with Thy-1 and immunoglobulin light chains. In the AD brain, the KPI-containing APP isoforms (APP751 and APP770) are upregulated [22], which may contribute to the impairment of metabolic enzymes and mitochondrial function in AD. In addition, cells expressing APP with or without the KPI domain (APP695, APP751) exhibit distinct susceptibilities to α- and β-secretase cleavage, which influences Aβ production [21]. In contrast to APP695, APP751 exhibits an increased transport to the plasma membrane due to its KPI domain situated in the extracellular domain, hence reducing the production of Aβ. In contrast, mutations in the KPI domain of APP751 lead to its retention in the endoplasmic reticulum (ER), which therefore stimulates the synthesis of Aβ [21]. The globular E1 domain, the AcD, and the helix-rich E2 domain are followed by the Aβ juxtamembrane sequence, which extends into the transmembrane domain of APP [23]. The E1 domain is broken into two regions: the HBD1 and the CuBD [24-26]. HBD1 is made up of a single α-helix and an anti-parallel β-sheet and contains a loop rich in heparin-binding basic residues. HBD1 is involved in neurite outgrowth and has been found to have a highly positively charged surface capable of interacting with glycosaminoglycans. The CuBD is a hydrophobic pocket immediately next to HBD1 that might serve as either a protein-binding site or a dimerization site [27].

**Figure 2.**
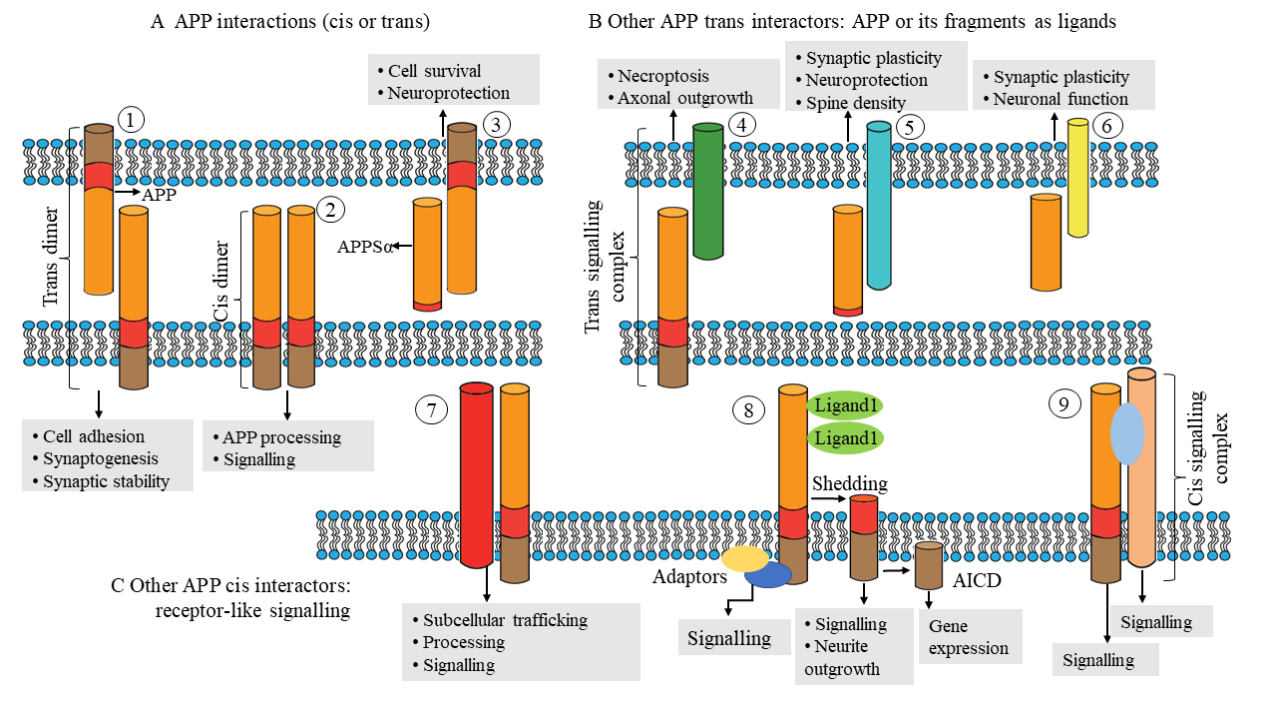
**The formation and functions of cis and trans dimers of APP**. The diagram depicts relationships between amyloid precursor protein (APP) family members (cis or trans). (**A**) Trans dimers ① and cis dimers ② can form homophilically, heterophilically, or between APP and an APP-like protein (APLP). sAPPα (α-secretase generated APP ectodomain fragment) interacts with transmembrane APP as an autocrine or paracrine ligand ③. (**B**) Other APP trans-interactors serve as receptors for APP and its fragments. For instance, membrane-bound APP ④ can bind to cell surface proteins such death receptor 6 (DR6) to stimulate signaling in adjacent cells. sAPPα interacts with undisclosed cell surface receptors as a ligand ⑤. Other secreted APP segments ⑥, such as Aη, may serve as ligands; however, only Aβ receptors have been discovered. (**C**) Other APP cis interactors are capable of cis signaling similar to that of receptors ⑦. for example, can activate caspases by interacting with the axonal membrane. Additionally, specific membrane proteins (such as members of the family of low-density lipoprotein receptors (LDLR) bind to APP and affect its subcellular distribution, processing, and internalization. APP may function as a receptor-like ligand-binding molecule ⑧. APP and C-terminal fragments (CTFs) can create signals via adaptors and can be processed to release the APP intracellular domain (AICD) for transcription regulation. APP can interact with larger membrane protein complexes ⑨ as coreceptors (for example, together with contactin).

In addition, CuBD may bind several metal ions. On the C-terminus of the E1 domain, the AcD is a hinge structure that is rich in glutamic acid and aspartic acid residues [28, 29]. Perhaps altering the relative location of the E1 domain may increase the binding of APP to other proteins [28]. The E2 domain is stiff and contains heparin-binding and metal-binding sites [30-32]. The E2 domain also contains the REMRS motif, which promotes cell proliferation and neurite extension and binds membrane-anchored heparan sulfate proteoglycans (HSPGs) [33, 34]. On the C-terminus of the E2 domain, Aβ is located partially in the extracellular/ juxtamembrane domain and partially in the transmembrane domain [35]. A GxxxG motif in the transmembrane domain has been associated with homodimerization and cholesterol binding [36, 37]. Human γ-secretase cleaves the transmembrane domains of APP into pathologically relevant Aβ [38]. Aβ peptides range in length from 39 to 42 amino acids, with the 42-amino-acid version (Aβ_42_) exhibiting reduced solubility [39]. Aβ aggregates to form oligomers, protofibrils, fibrils, and plaques, which are pathological hallmarks of AD [40]. Aβ buildup in the brain is regarded as the first step in the progression of AD [41]. Aβ formation occurs all the time in the brain, while Aβ aggregation/deposition begins mostly in the hippocampus and entorhinal cortex. The APP intracellular domain (AICD) [42] is next to the Aβ sequence and contains phosphorylation sites and a YENPTY sorting motif [25, 43]. AICD is the C-terminal fragment (CTF) of APP released after γ-secretase cleavage of the CTFs. This fragment is generated by both amyloidogenic and nonamyloidogenic routes in addition to a number of other APP cleavage mechanisms. AICD enhances cell death, stimulates tau phosphorylation, reduces neuronal activity, and influences calcium homeostasis, consequently reducing neuronal susceptibility to stimuli and supporting a negative effect [44, 45]. AICD can vary in length, with AICD_1-59_ and AICD_1-57_ being the most prevalent variants. AICD_1-57_ is believed to induce cell death more effectively than AICD_1-59_ [46]. The finding that AD brain samples from both familial and sporadic patients have higher AICD levels than age-matched controls suggests that the accumulation of AICD may play a role in the pathogenesis of AD [47]. Glycogen synthase kinase-3β is activated by increased AICD and thereafter phosphorylates tau [48, 49]. Overall, APP and its isoforms play important roles in the pathogenesis of AD through the production and aggregation of Aβ, and the accumulation of the intracellular domain fragment AICD may also contribute to the development of AD.

## 3. Functions of the APP family members

Members of the APP family are truly multimodal proteins that influence biological processes ranging from transcriptional control to synaptic activity. They can function as receptor-like proteins on the cell surface (Fig. 2A) or as ligands (Fig. 2B, 2C), mediating their actions either from the cell surface or via their released proteolytic fragments.

### 3.1 Formation and functions of cis and trans dimers

The APP family consists of several members, including APP and amyloid precursor-like proteins (APLPs), which can form homotypic or heterotypic cis dimers mostly via the E1 and E2 domains, although heparin binding can also regulate dimer formation [50]. Importantly, APP and APLPs may also form trans dimers, allowing them to serve as synaptic adhesion molecules in vitro [51] and at the neuromuscular junction (NMJ) in vivo [52]. On the axons of neurons cocultured with human embryonic kidney cells expressing APP, presynaptic differentiations known as hemisynapses have been found[53]. As transsynaptic adhesion is dependent on the pool of APP on the cell surface, APP variants with extracellular secretase site mutations exhibit higher synaptogenic activity [54]. Copper-binding sites in the growth factor-like domain that are conserved in APP and APLP1 but not in APLP2 are required for the induction of presynaptic specialization by APP [55]. Furthermore, α-secretase is an enzyme that cleaves APP in the extracellular domain, which releases a soluble fragment called soluble APP α (sAPPα) [56]. sAPPα can bind to cell-surface APP as a ligand and then promotes neuroprotection through Gαo protein signaling [57, 58]. The homotypic and heterotypic dimerization of members of the APP family, their transsynaptic adhesion, copper-binding sites, and enzymatic activity have been linked to in the pathogenesis of AD.

### 3.2 Other APP family interactors and their roles in signaling

**Figure 3.**
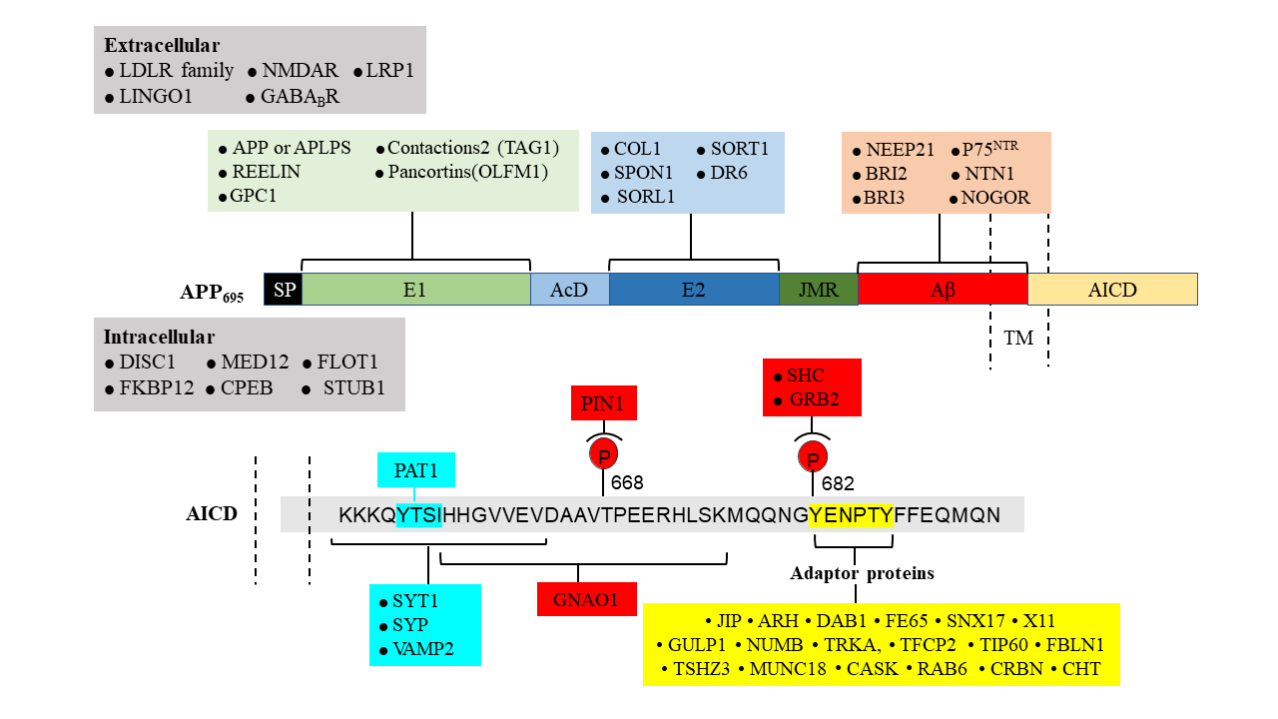
**Interactors and interaction sites of APP**. The extracellular domain (top) and intracellular domain (bottom) of APP interact with several proteins. Known interactors with mapped APP-binding motifs are illustrated with the same color as their respective APP domains. LRP1, the LDLR family, NMDAR, LINGO1 and GABA_B_R members have been found to interact extracellularly with APP. LRP1, Low density lipoprotein receptor-related protein 1; LDLR family, LDLR-related protein; NMDAR, NMDA receptor; LINGO1, leucine-rich repeat and Ig domain-containing Nogo receptor-interacting protein 1; GABA_B_R, GABA type B receptor; SP, signal peptide; E1, E1 region; REELN, reelin protein ; GPC1, glypican 1; OLFM1, olfactomedin 1; TAG1, transient axonal glycoprotein 1 (also known as contactin 2); AcD, acidic domain; E2, E2 region; COL1, collagen type 1; SORT1, sortilin 1; SPON1, F-spondin; SORL1, sortilin-related receptor; DR6, death receptor 6; JMR, juxtamembrane region; Aβ, β-amyloid peptides; NEEP21, neuron-enriched endosomal protein of 21 kDa; BRI2, brichos domain-containing 2; BRI3, brichos domain-containing 3; P75^NTR^, low affinity neurotrophin receptor p75; NTN1, netrin 1; NOGOR, Nogo-66 receptor; DISC1, MED12, CPEB, FLOT1, CHT, FKBP12 and STUB1 have been found to interact extracellularly with APP. DISC1, disrupted in schizophrenia 1 protein; MED12, Mediator complex subunit 12; CPEB, cytoplasmic polyadenylation element-binding protein; FLOT1, flotilin 1; FKBP12, FK506 binding protein 12; STUB1, STIP1 homology and U box-containing protein 1. The bottom panel reveals binding proteins in the same color as their interaction motifs, which are designated by square brackets or bold text inside the AICD sequence. PAT1, protein interacting with APP tail 1; PIN1, peptidyl-prolyl cis-trans isomerase NIMA-interacting 1; SHC, SRC homology 2 domain-containing-transforming protein 1; GRB2, growth factor receptor-bound protein 2; SYT1, synaptotagmin 1; SYP, synaptophysin; VAMP2, vesicle-associated membrane protein 2. GNAO1 encodes Gαo, the α subunit of Go, a member of the Gi/o family of heterotrimeric G protein signal transducers. Adaptor proteins; JIP, JUN N-terminal kinase-interacting protein; ARH, autosomal recessive hypercholesterolemia protein; DAB1, disabled homolog 1; FE65, The scaffolding protein family Fe65 was identified as an interaction partner of the amyloid precursor protein (APP); SNX17, sorting nexin 17; X11 protein family are multidomain proteins composed of a conserved PTB domain and two C-terminal PDZ domains; NUMB, a key regulator of cell fate, TRKA, tyrosine kinase receptor A; TFCP2, transcription factor CP2 (also known as LBP2/CP2/LSF); TIP60, the founding member of MYST histone acetyltransferase family; Tshz3,Teashirt-3 is expressed in smooth muscle cell precursors; MUNC18, regulating neurosynaptic plasticity, neurodevelopment and neuroendocrine cell release functions; CASK, Calcium/calmodulin-dependent serine protein kinase; Rab6, small GTP-binding protein; SNAREs, soluble N-ethylmaleimide-sensitive factor attachment protein receptors; NOTCH, Notch signaling in the control of neurogenesis and regeneration in the embryo and adult; CRBN, protein cerebron; CHT, high-affinity choline transporter; FBLN1, fib

While APP family members lack enzymatic activity, signal transmission to downstream signaling pathways is dependent on interactions with other membrane proteins and/or adaptors [59]. To date, more than 200 extracellular and intracellular binding partners have been identified [60]. The extracellular and cytoplasmic regions of APP have essential cell adhesion molecule (CAM) characteristics (Fig. 3).

Similar to adhesion proteins such as cadherins, integrin β1 and L1, these properties suggest that APP may serve as a CAM capable of linking the extracellular environment to the cytoskeleton via scaffold protein(s) [61]. By interacting with multiple extracellular matrix (ECM) components and signaling through scaffolding proteins, APP may coordinate the plastic remodeling of cell adhesions and the dynamic architecture of the subcortical cytoskeleton [62]. APP binds several ECM proteins, playing crucial roles in the formation and maintenance of brain architecture throughout adulthood [63]. APP's ability to bind diverse ECM components may enable migratory neurons to adapt to a complex neurodevelopmental environment [63]. APP interacts with important in vivo interactors include brichos domain-containing 2 (BRI2) and BRI3 [64], which inhibit APP cleavage by α-, β-, and γ-secretases. Mutations in ITM2B (which encodes the BRI2 precursor protein) that are associated with rare familial Danish and British dementias resembling AD result in decreased BRI2 protein levels and, consequently, increased APP processing [65]. APP interacts with ECM proteins (such as collagen and heparin) and HSPGs via its HBDs (including glypican and syndecan). APP interacts directly or through adaptors with a variety of transmembrane proteins, including members of the lipoprotein receptor family such as low-density lipoprotein receptor-related protein 4, a crucial organizer of synaptogenesis at the NMJ [66]. APP may also operate as a coreceptor to enhance the target-specific arborization of retinotectal axons [67]. The E2 domain can create either cis or trans connections with a member of the tumor necrosis factor receptor family, death receptor 6 (DR6) [68]. Cis APP-DR6 interactions on the axonal membrane can generate caspases that are implicated in experience-dependent axonal plasticity, especially whisker-induced axonal pruning [69].

Previous research has suggested that axonal pruning of developing sensory neurons requires a secretase-generated soluble N-terminal APP fragment (N-APPs_1-286_). Notably, an APP-DR6 interaction has been hypothesized to contribute to neurodegeneration associated with AD [68]. In two transgenic AD mouse models, silencing of the DR6 gene was found to have no effect on amyloid plaque production, gliosis, synaptic loss, or cognitive impairment [70]. Reelin has been demonstrated to raise the surface levels of APP, hence inhibiting APP endocytosis and β-secretase processing. Moreover, Reelin is an extracellular protein involved in neuronal migration during cortical development that is released by Cajal-Retzius cells [71]. One study revealed synergistic action among APP, Reelin, and integrins that increases neurite outgrowth and dendritic arborization [52]. APP also binds the protein F-Spondin, which is involved in cell–cell communication, axonal elongation, and pathfinding, and inhibits the secretase cleavage of APP [72]. Additionally, it has been postulated that the connection between APP and F-Spondin helps suppress cell adhesion by encouraging contact with other substrates [73]. Laminin is another essential ECM protein that binds APP during neurodevelopment and is found in brain tissue [74]. APP is present and abundant in growth cone (GC) adhesions on laminin with integrin, CD81, and focal adhesion kinase (FAK). This discovery is consistent with the significant colocalization of APP and integrins in the axonal GCs of hippocampal neurons in mice, particularly in the lamellipodia and filopodia, where the dynamic actin cytoskeleton is present [75]. APP dose impacts adhesion and axonal outgrowth in cultured hippocampal neurons grown on laminin. HSPGs are additional ECM components [76]. Previous in vitro studies using chick sympathetic neurons and mouse hippocampal neurons have revealed that APP is able to bind unique kinds of HSPGs produced during neurodevelopment to promote neurite outgrowth [77]. The extracellular portion of the neurotrophic factor receptor p75^NTR^ also contains an APP binding site [78]. Comparatively, the location of the p75^NTR^ binding site on Aβ is at amino acids. Extracellular p75^NTR^ inhibits Aβ aggregation and encourages Aβ fibril depolymerization. Intracerebral injection of p75^NTR^ can effectively eliminate local Aβ deposition [79]. p75^NTR^ is a neurotrophic receptor for growth factors and plays an essential role in neural development and plasticity, and the interaction with APP suggests a potential mechanism for regulating Aβ aggregation and modulating neurotrophic signaling in response to injury or disease [78]. Moreover, p75^NTR^ can activate signaling pathways that promote neuronal survival, neurite outgrowth, and synaptic plasticity, indicating its critical role in the nervous system [80]. Intriguingly, proteomic analyses of the presynaptic active zone have revealed that APP and APLPs interact with a variety of synaptic proteins, including bassoon, synaptophysin, and SNAP receptor (SNARE) complex proteins, either directly via their C-termini or indirectly via X11 adapter proteins [81]. The multitude of extracellular and intracellular binding partners of APP, which include extracellular matrix components, scaffold proteins, and transmembrane proteins, as well as its involvement in neuronal adhesion and migration, suggest the importance of studying the interactions and enzymatic activity of the APP family in relation to AD.

### 3.3 Subcellular localization and regulation of APP processing

Members of the APP family have distinct cell-surface levels, and the location of APP inside the cell alters its processing by transmembrane secretases [82] (Fig. 5). The accumulation of APP on the surface favors nonamyloidogenic processing, whereas preservation of the presence of APP in acidic compartments, such as early endosomes, promotes amyloidogenesis [83]. Either α-secretase cleaves APP to liberate sAPPα or APP is reinternalized to the endosome at the cell surface[84]. Consequently, the surface pool of APP is the outcome of secretory trafficking, internalization, and the processing efficiency of secretases. APP and APLPs are found in the somatodendritic and axonal compartments of neurons. In axons, they are largely transported in vesicles distinct from those detected by synaptophysin staining, which become enriched in active zones [85]. APP and APLPs arise via the secretory pathway and are abundant in intracellular membrane compartments prior to reaching the cell surface. APP is highly localized at the cell surface, and vesicles containing APP and β-secretase are spatially separated inside dendrites, limiting their physical proximity [86]. Neuronal activity nonetheless encourages the convergence of APP and secretase in recycling endosomes as one of the early steps in Aβ production, which involves endosomes and other intracellular organelles. The C-terminus of APP is a signaling center with multiple protein-interaction motifs (Fig. 5). The YENPTY motif is involved in clathrin-mediated endocytosis, influences APP processing, and interacts with several phosphotyrosine-binding domain-containing proteins, including the Jip-1 and FE65 adaptor families [87]. The phosphorylation status of Thr668 and Tyr682 that regulate the APP interactome by either establishing a docking site (for SH2-domain-containing proteins, such as SRC kinase family members) or inhibiting protein interactions (for example, of FE65 family members) [88]. When APP CTFs accumulate in the absence of γ-secretase activity, in vitro and in vivo axodendritic growth may be enhanced [89]. Furthermore, the interaction of APP family members with the NMDA receptor (NMDAR) may promote NMDAR cell surface localization [90]. In vitro, membrane-tethered AICD overexpression stimulates neurite outgrowth via a Gαs protein-adenylate cyclase-cAMP cascade [91]. AICD, which is released by γ-secretase processing, has been demonstrated to translocate into the nucleus and may regulate transcription [92]. This has been regularly demonstrated in vitro, particularly in AICD-overexpressing cells, but research on the physiological expression levels of AICD and in vivo research have produced contradictory results. The very unstable nature of AICD makes its identification difficult. Recent studies have provided new information on the activities of AICD in regulating transcription [43, 44, 93, 94]. For example, CBP is a coactivator of transcription that regulates the expression of many genes involved in synaptic plasticity and long-term memory formation [95, 96]. Fe65 is a transcriptional coregulator that interacts with AICD to modulate FOXO3a gene expression [93, 97], and Tip60 is a histone acetyltransferase that plays a key role in regulating gene expression in response to neuronal activity [98]. One consequence of restoring normal AICD function in neurons could be improved cognitive function [99]. In conditions such as AD, AICD function is disrupted, which can lead to impaired neuronal function and cognitive decline [15]. Therefore, any attempts to restore normal AICD function would need to be carefully evaluated and may not be appropriate in all cases [100, 101]. The distinct cell-surface levels of APP and its processing by transmembrane secretases, as well as its presence in different neuronal compartments, the interactions of its C-terminus with multiple protein-interaction motifs, and the potential role of AICD in regulating transcription, suggest the importance of studying the molecular mechanisms underlying APP function and its contribution to AD pathogenesis.

**Table 2:** Functions of APP fragments in the mammalian CNS.

APP fragment	Functions and effects	Reference
**sAPPα**	LTP and NMDAR currents in DG of anesthetized rats;	[220-222]
	**Rescues spine density of APP organotypic hippocampal cultures; rescues LTP and spatial learning in aged APP mice;**	[223, 224]
	**Tg OE in APP/PS1 mice inhibits the amyloidogenic pathway, reduces plaque deposition and reduces GSK3β-dependent tau phosphorylation;**	[225]
	**Protects against TBI; neuronal death during transient ischemia; and hypoxia in acute hippocampal slices; Stimulates aduit neurogenesis at the subventricular zone;**	[226, 227]
**sAPPβ**	Stable metabolite in vivo, not associated with increased cell death, induces transcription of transthyretin and klotho;	[228, 229]
Aβ*	Generated by meprin cleavage, High aggregation propensity: potential seed for Aβ deposition;	[115, 117]
**p3**	Physiological or trophic function unknown; no pathological effects reported;	[230]
**Aη-α**	Neuronal activity and LTP in wild-type hippocampal slices, Upregulated upon β-secretae inhibition;	[231]
**Aη-β**	None of the pathological properties reported;	[232]
**APPsη**	Physiological function unknown;	[233]
**APPsσ**	Tg AD model mice that also lack σ-secretase show reduced Aβ load and ameliorated functional deficits;	[234]
sAPPβ*	Generated by meprin cleavage, physiological function unknown;	[235]
**CTFα**	Physiological function unknown;	[236]
**CTFβ**	Injection of CTFβ impairs working memory and induces neurodegeneration and gliosis; Tg CTFβ OE induces neurodegeneration, reduces LTP and impairs cognition, impairs lysosomal autophagic function;	[237]
**CTFη**	Associated with plaques. upregulated upon β-secretase inhibition;	[118]
**C31**	C31 complexes with APP to recruit the interacting partners that initiate the signals related to cellular toxicity;	[238, 239]
**Jcasp**	Intracelular delivery to acute hippocampal slices reduces basal synaptic transmission, increases PPF and synaptic frequency facilitation in wild-type, but not in APP, mice, and reduces the rate;	[239, 240]

Aβ, amyloid; Aβ*_2-x_, the peptide beginning with amino acid 2 of Aβ; AD, Alzheimer's disease. AICD, APP intracellular domain; sAPPβ*, one amino acid longer than sAPPβ generated by β-secretase cleavage; CTF, C-terminal fragment; DG, dentate gyrus; APP, amyloid precursor protein; CTF nAChR, nicotinic acetylcholine receptor; GSK3, glycogen synthase kinase 3; NMDAR, NMDA receptor; OE, overexpression; PTP, posttetanic potentiation; PPF, paired-pulse facilitation; LTP, long-term potentiation; Tg, transgenic; TBI, traumatic brain injury

## 4. APP processing pathways

There are two primary pathways for processing APP: amyloidogenic and nonamyloidogenic processing [102, 103]. The processing pathway depends on the colocalization of the protein and secretases [104]. Altered APP processing by secretases, as indicated by increased in Aβ production and changes in the Aβ_40_/Aβ_42_ ratio, has been demonstrated to occur in both AD patients' primary cells and transgenic AD mouse models [66].

### 4.1 Canonical APP processing

**Figure 4.**
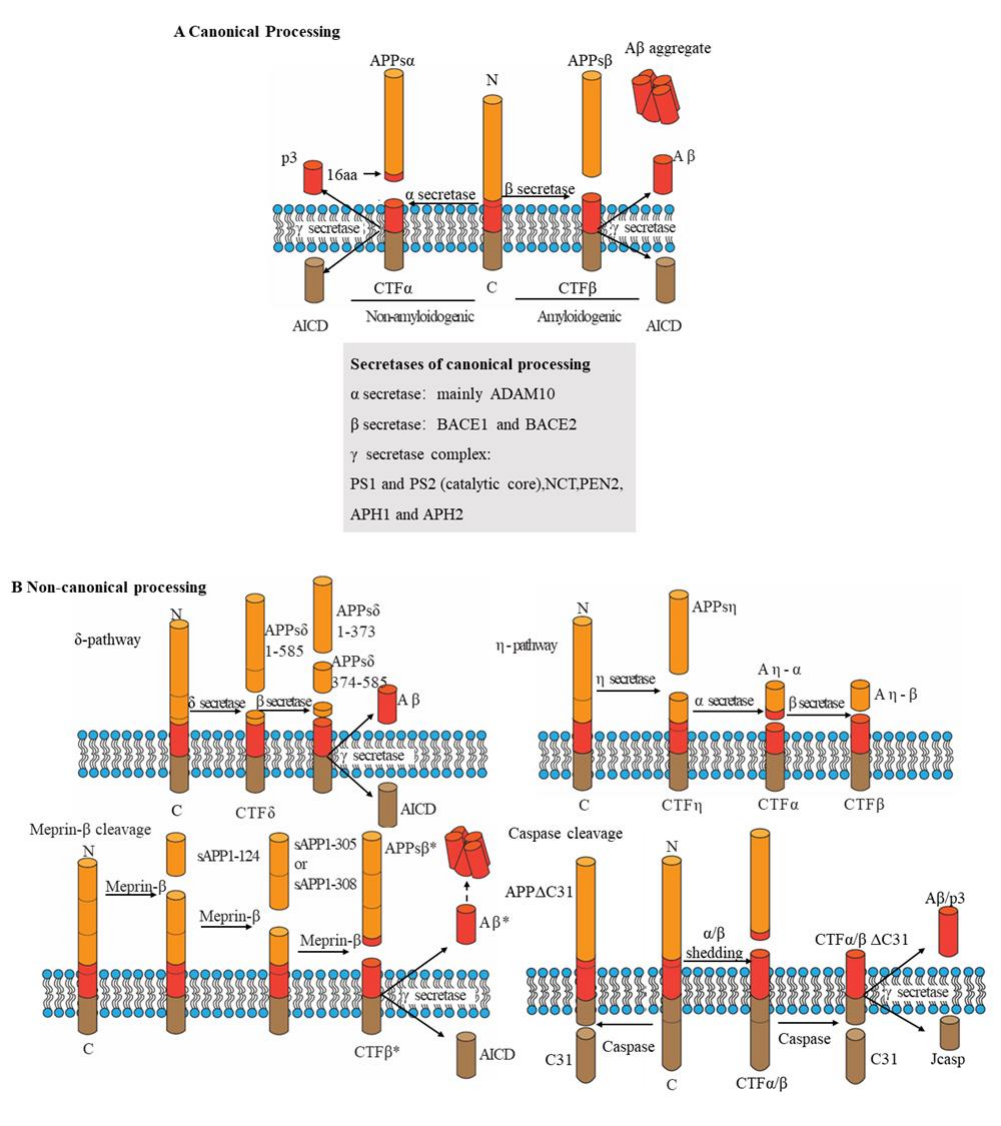
**Schematic overview of APP-processing pathways**. (**A**) The graphic displays conventional amyloid precursor protein manufacturing routes (APP). In the amyloid (Aβ) region (left), processing by secretase along the nonamyloidogenic pathway releases sAPPα (α-secretase-generated APP ectodomain fragment) and generates p3. The amyloidogenic pathway (right) produces Aβ (through β- and γ-secretase cleavage) and sAPPβ. An intracellular fragment (APP intracellular domain (AICD)) is released during both procedures. The locations of areas of cleavage are provided. B The graphic illustrates nonstandard APP processing. Secretase cleaves APPs into three soluble fragments and a C-terminal fragment-δ (CTFδ) that is subsequently processed by β- and γ secretase (top left panel). Three sites are utilized by Meprin β- and γ-secretase to form three soluble fragments (lower left panel). CTFβ* is one amino acid residue shorter than CTFβ and is generated after the release of sAPPβ*. Aβ* denotes the amino-terminally shortened version of Aβ_2-x_. By secretase cleavage, soluble APPsη and CTFη are produced, which are subsequently further processed by α-secretase or β-secretase to form Aηα or Aηβ (top right panel). Caspases cleave within the intracellular domain to form C31, which is then cleaved by secretases to yield Jcasp (lower right panel). (**B**) The diagram illustrates Aβ-generating and N-terminal cleavage sites. The numbers represent the C-terminal residues of full-length APP695 and the processing sites for APP. The upper panel depicts the positions of canonical secretases, with the residues targeted by γ-secretase numbered in relation to the Aβ N-terminal. The locations of noncanonical processing are depicted in the bottom panels. APH1, anterior pharynx defective 1; NCT, nicastrin; PEN2, presenilin enhancer 2; ADAM10, disintegrin and metalloproteinase domain-containing protein 10; PEN2, presenilin enhancer 2.

The amyloidogenic pathway begins with endosomal internalization of APP (Fig. 4A and Table 2) [105]. β-Secretase on the endosomal membrane cleaves APP into two forms: a soluble APP fragment (sAPPβ) and a membrane-anchored carboxyl terminal fragment (CTFβ or C99) within the lipid bilayer. CTFβs further cleaved by γ-secretase to generate Aβ monomers and AICD [106]. The Aβ cleavage products consisting of 43-51 amino acids are further cleaved into the Aβ_40_ and Aβ_42_ forms [107]. Multiple investigations have demonstrated that Aβ peptides are generated at separate places inside the cell. Secreted Aβ peptides are discharged outside of the cell in exosomes, where they form oligomers, protofibrils, fibrils, and eventually senile plaques (SPs). Nonamyloidogenic processing of APP begins in the plasma membrane, where α-secretase resides. APP is cleaved at the cell surface by α-secretase, resulting in the release of sAPPα and a carboxyl terminal fragment containing 83 amino acids (CTFα or C83). CTFα can be internalized and then processed by γ-secretase in endosomes to generate p3 (3 kDa) and AICD [106].

### 4.2 Noncanonical APP processing

In addition to the well-known APP secretases, recent studies have identified other proteases that contribute to the production of Aβ species (Fig. 4B and Table 2). The potential involvement of these proteases in neurodegeneration may open up new avenues for AD treatment research [108]. Cathepsin B, a lysosomal cysteine protease that is a novel β-secretase, was discovered for the first time in the SPs of postmortem AD brains [109]. A series of in vivo studies conducted by one group revealed that inhibition of cathepsin B by its various inhibitors or genetic deletion of cathepsin B decreases Aβ levels and improves memory function. Moreover, plasma cathepsin B levels have been found to be higher in AD patients than in healthy controls. In a recent study, hippocampal injections of adeno-associated viruses expressing cathepsin B into APP/PS1 transgenic mice reduced amyloidogenesis and enhanced learning and behavioral functions [110]. However, it has also been demonstrated that cathepsin B plays a critical role in inducing microglia-mediated neuroinflammation in neurons upon systemic exposure of mice to lipopolysaccharide from periodontal bacteria. Gum disease is a chronic inflammatory condition caused by bacterial accumulation in dental plaque, and chronic inflammation may be linked to AD [111]. The inflammation and bacterial products associated with gum disease can travel to the brain and trigger inflammation, which may contribute to the accumulation of amyloid plaques and tau tangles in the brain, hallmark features of AD [112]. For example, *Porphyromonas gingivalis* is found in AD brains, suggesting that cathepsin B may be a potential therapeutic target for the treatment of periodontitis-associated AD [113, 114]. These conflicting findings strongly suggest the need for further investigation into the involvement of cathepsin B in amyloid disease. Meprin β, a zinc-dependent metalloprotease present in several tissues, is another enzyme that cleaves APP [115]. Meprin β generates N-terminal APP fragments of approximately 11 and 20 kDa beginning with the first or second amino acid residue of Aβ by cleaving APP at Met596-Asp597, Asp597-Ala598, and Ala598-Glu599 [116]. Meprin β, also known as alternative β-secretase, has greater activity for APP than β-site APP cleaving enzyme 1 (BACE1) and is independent of BACE1/2 [117]. CTF-η is generated mostly by η-secretase cleavage at APP695 amino acids 504-505 [118]. ADAM10 and BACE1 release long (Aηα) and short (Aηβ) Aη peptides through their continued digestion of CTF-η (C191). CTF-η production was discovered to be partially mediated by membrane-bound matrix metalloproteinase (MT5-MMP), which has previously been demonstrated to cleave APP in vitro [118]. In vivo investigations have demonstrated that the level of CTF-η is elevated in both AD model mice (APP/PS1) and AD human brains [119]. In addition to MT5-MMP, MT1-MMP has recently been characterized as proamyloidogenic [120]. In vitro, MT1-MMP enhances Aβ and CTFβ (or C99 produced following BACE1 APP cleavage) synthesis through APP trafficking regulation [121]. δ-Secretase, an age-dependent asparagine endopeptidase, cleaves APP695 at Asn373 and Asn585 residues on the extracellular motif as well as tau, another actor in AD pathogenesis [122, 123]. δ-Secretase appears to interact with APP in endosomes, and δ-secretase-mediated APP cleavage enhances the subsequent processing of APP by BACE1, hence increasing Aβ production [123]. During apoptosis, caspases (mostly caspase-3) can directly cleave APP farther downstream at position Asp664 (based on the APP695 sequence) within the cytoplasmic tail to release a fragment containing the final 31 amino acids of APP (called C31) [13, 124]. Additional γ-cleavage forms the Jcasp fragment, which contains the area between the γ- and caspase-cleavage sites [125]. C31 combines with APP to recruit the interaction partners that begin signals associated with cellular toxicity, which is one probable explanation for C31's toxicity [126, 127]. Compared to C31, Jcasp appears to serve a small function in cytotoxicity.

## 5. Regulation of APP processing

**Figure 5.**
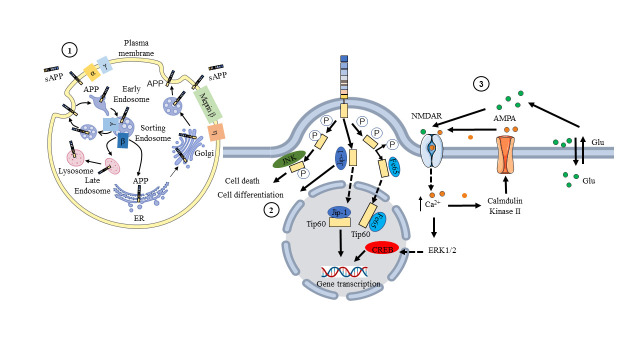
**Cellular regulation and metabolism of APP**. ① The digestive and metabolic cycle of APP is secreted via the endocytosis system of intracellular cells; ② A diagram illustrating the intracellular pathway of AICD. Phosphorylated AICD interacts with JNK, which leads to cell death, with JIP, which leads to cell differentiation, and with Fe65 or JIP, which leads to nuclear transport and regulation of gene transcription (NMDAR). ③ Calcium ions (Ca^2+^) and glutamate (Glu) together activate NMDA receptors (NMDAR). NMDAR receptor activation enhances membrane expression of AMPA receptors (AMPARs) and activates nuclear transcription factors.

Upon the cleavage of the signal peptide by the ER, APP is processed through the secretory pathway and transported via the Golgi apparatus to the plasma membrane, where it predominantly localizes [128-130]. Upon endocytosis, APP is targeted to early endosomes and subsequently sorted to three distinct pathways (Fig. 5): (I) a subset of APP molecules undergoes recycling to the cell surface [119, 131], (II) a different fraction of APP is transported retrogradely from endosomes back to the Trans-Golgi Network via a retromer-mediated pathway [132], and (III) some APP molecules are targeted to late endosomes, which fuse with lysosomes where APP is degraded. APP is primarily endocytosed by clathrin-coated vesicles into early endosomes [133, 134], and its internalization is also dependent on cholesterol, indicating that clathrin- and cholesterol-dependent endocytosis overlap [135, 136]. Other transmembrane proteins, including low-density lipoprotein receptors (LDLRs), Vps10p-Doamin (Vps10p-D) receptors, and calsyntenins, can influence APP intracellular transit and processing [137-139]. LDLRs endocytose ligands that are carried to endosomes and recycling or lysosomal compartments, while the receptors are returned to the plasma membrane [140]. Sortilin-receptor with A-type repeats (SorLA) is a unique mosaic receptor [141, 142] that combines structural elements of the LDLR family by containing EGF-type and ligand-binding repeats with the N-terminal Vps10p-D that is characteristic of the Vps10p-D receptor family [143]. Calsyntenins are the third category of type-I transmembrane proteins mentioned in this review that modulate APP transport (calsyntenin 1-3, Clstn 1-3). These were initially isolated as calcium-binding proteins in postsynaptic neurons [144] and have also been designated Alcadein. Calsyntenin-1 and -2 both contain a C-terminal calcium-binding site, which is mediated through an acidic amino acid stretch within APP. Notably, this stretch is shorter in calsyntenin-2 than in calsyntenin-1, which may have implications for their respective calcium-binding properties and downstream signaling functions [144-146].

## 6. Mutations in APP

APP mutations can be categorized based on their effect on the expression levels of the various Aβ fragments [147] (Fig. 6 and Table 3). Progress in AD genetics began with the identification of autosomal dominant mutations in *APP*. Currently, there are 75 known single nucleotide mutations in APP that lead to (https://www.alzforum.org). APP mutations such as Swedish, p. Glu693Gly, p. KM670/671NL, and p. V717I have an impact on Aβ production and contribute to AD pathogenesis by increasing Aβ aggregation and plaque formation, cerebral amyloid angiopathy, and cognitive decline [148]. The protective p.A673T substitution reduces amyloidogenic peptide formation and supports the hypothesis of reducing β-cleavage to prevent AD [149]. Methylation of the APP promoter by dCas9-Dnmt3a decreases neurotoxic Aβ peptides and improves learning and memory impairment in AD [150]. Individuals with DS and duplications of small segments of chromosome 21 containing an additional copy of APP support the idea that alterations in the amount or sequence of Aβ are sufficient to cause AD, with multimeric Aβ being critical for pathogenesis [148]. APP N-terminal disease-associated mutations involve the HBD and potentially also affect interactions of full-length APP, sAPPα and sAPPβ, with consequences for disease progression in addition to any effects caused by the changes in cleavage or behavior of Aβ. The APP S198P mutation in the N-terminal region of APP has been found to increase amyloidosis in cultured cells and transgenic mouse models [151]. The introduction of the S198P mutation appears to accelerate the folding and transfer of APP from the ER to the β-secretase active compartment [151]. Moreover, in one report, Japanese siblings with a novel N-terminal V225A mutation in the AcD domain of the APP gene did not appear to have significantly abnormal Aβ levels in cerebrospinal fluid but had significantly increased extracellular tau protein levels. However, these siblings developed progressive dementia at age 57 with Aβ and tau lesions [152]. Presumably, APP can also assist in the diffusion and transport of pathological tau protein, thereby inducing tau pathological changes in AD. The significance of APP mutations in the development of AD is still an active area of research, and how these mutations contribute to disease pathogenesis is not yet fully understood. However, understanding the effects of APP mutations on Aβ production and aggregation is an important step toward developing effective treatments for AD.

**Table 3:** Mutations in APP.

Mutations	Biological Effects	Areas	Group	Ref.
**A673V, E682K (Leuven);**	Shifts β-secretase processing of APP toward the amyloidogenic pathway and increases Aβ aggregation	JMR	1	[99,100]
**K687N;**	Reduces APP cleavage by α-secretase; reduced production of total sAPP and sAPPα; increased Aβ_42_ /Aβ_40_	JMR	1	[101]
**A235V, Y538H, P620L, A673T (Icelandic), E693del (Osaka) T719P, A713V, V715M (French); A692G(Flemish), D694N(lowa);**	Decreased total Aβ in cells, without altering Aβ_42_ /Aβ_40_ ratio	AcD, JMR, TM	2/3/X	[102-112]
**KM670/671NL, P620L, S198P;**	Increased total Aβ in cells, without altering Aβ_42_ /Aβ_40_ ratio	AcD, JMR	1/X	[102-103,115]
**P620A, G708G, H733P;**	Increased Aβ42, without altering Aβ_42_ /Aβ_40_ ratio	JMR, TM, ACID	3/X	[112-114]
**S614G, A713T, V715M(French), T714I (Austrian), V715A(German), V717F (Indiana);**	Increased Aβ_42_/Aβ_40_ ratio	JMR, TM	3/X	[112,115-117]
**E693K (Italian);**	Reduced Aβ_42_ /Aβ_40_ ratio; decreased Aβ_42_ comparable Aβ_40_ to wild-type APP	JMR	2	[110]
**P620A, D678H (Taiwanese), T714I (Austrian), E682K (Leuven), I716F I716V (Florida), V717I (London), V717G, T719N, M722K, L723P (Australian), K724N (Belgian);**	Increased Aβ_42_ and the Aβ_42_ /Aβ_40_ ratio in cells	JMR, TM, ACID	1/3/X	[104-106, 111, 120, 121]
**H677R (English), D678N(Tottori), E693Q (Dutch), D694N (Iowa), E693G (Arctic);**	Accelerated oligomerization kinetics and greater cytotoxicity than wild-type Aβ	JMR	2/3	[127,117-120]
**F690_V695del (Uppsala deletion);**	Appears to largely eliminate nonamyloidogenic processing of APP and leads to the generation of rapidly aggregating Aβ peptides lacking amino acids 19-24	JMR	X	[133]
**APP promoter**	Promoter mutations leading to increased APP levels share some features with DS; may vary between specific mutations		X	[135]
**Down Syndrome (DS)**	Increased Aβ oligomers; complex changes in levels of Aβ species in plasma and CSF; levels of Aβ_40_ while initially higher in DS than normal controls; levels of Aβ_42_ and Aβ_42_ /Aβ_40_ are initially lower but increase with DS dementia		X	[136]

Aβ, amyloid β; AD, Alzheimer’s disease; APP, amyloid precursor protein; CAA, cerebral amyloid angiopathy; CSF, cerebrospinal fluid; DS, Down syndrome; FAD, forms of AD; IDE, insulin degrading enzyme; MRI, magnetic resonance imaging; NFT, neurofibrillary tangle; SAD, sporadic AD. Areas represent the domain in the app where the mutation occurred. Group 1 features increased total Aβ, Aβ40, and Aβ42 levels and an increased Aβ42/Aβ40 ratio and is associated with mutations around the α-secretase site. Group 2 features reduced total Aβ, Aβ40, and Aβ42 levels and a reduced Aβ42/Aβ40 ratio. Group 3 exhibited reduced total Aβ and Aβ40 levels combined with increased Aβ42 levels and an increased Aβ42/Aβ40 ratio, and the mutations were associated with the γ-secretase site. Group X contains mutations that cannot be otherwise grouped because of a lack of data. Detailed descriptions are not available for recently discovered mutations, as individuals have not yet come to autopsy.

**Table 4:** Stage of clinical development of anti-APP drugs to treat Alzheimer’s disease.

Drug target	Phase I	Phase II	Phase III	Approved
**α-Secretase activators**	N/A	Acitretin	N/A	N/A
**BACE inhibitors**	N/A	N/A	N/A	N/A
**γ-secretase inhibitors and modulators**	N/A	NIC5-15	N/A	N/A
**Aβ aggregation inhibitors**	Contraloid	CT1812 PBT2	ALZ-801 ALZT-OP1 Simufilam	N/A
**Passive immunotherapy**	ACU193,DNL919 IBC-Ab002, MEDI1814,PRX012, Trontinemab	ABBV-916	Donanemab Remternetug Solanezumab	Aducanumab Lecanemab-irmb
**Active immunotherapy**	ALZ-101 AV-1959D	ABvac40 ACI-24, UB-311	N/A	N/A
**Inhibit APP protein synthesis**	N/A	Posiphen	N/A	N/A

The table does not include drugs that indirectly interfere with amyloid-β (APP) or do not have fully proven anti-APP mechanisms of action.

## 7. APP-targeted treatment strategies in AD

### 7.1 Immunotherapy

**Figure 7.**
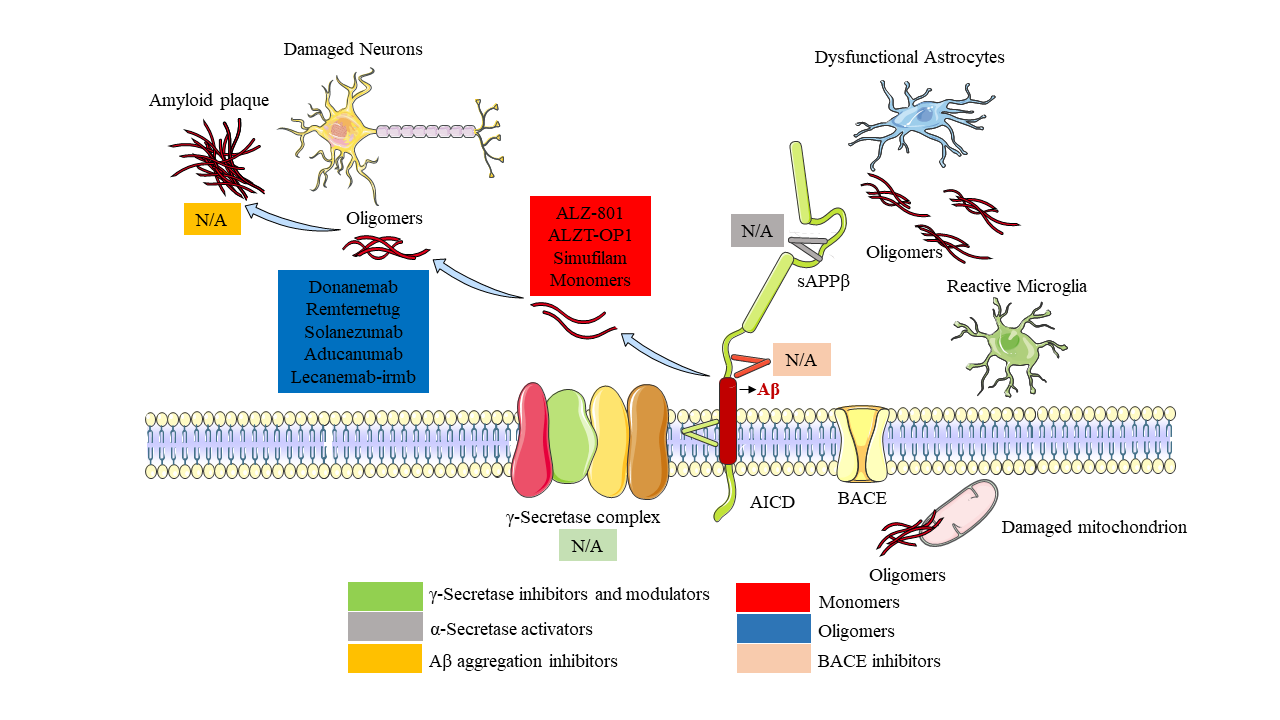
**APP as a therapeutic target for AD**. Mechanisms of action of the main anti-APP drugs that are currently in Phase III/Approved for the treatment of Alzheimer disease. AICD, amyloid precursor protein intracellular domain; BACE, β-secretase; sAPPβ, soluble amyloid precursor protein-β.

Immunotherapy is the most touted technique for decreasing Aβ levels (Fig. 7 and Table 4). This strategy relies mostly on boosting immune cells such as B and T lymphocytes and on activating microglia to increase their phagocytic capability [153]. Immunotherapy treatments reduce the extracellular levels of proinflammatory antigens, stimulate the microglial clearance of toxic aggregates, and attenuate potentially damaging microglial inflammatory responses, resulting in neuroprotective benefits that delay the progression of the illness [154]. Recent trials of aducanumab and lecanemab have demonstrated efficacy in reducing Aβ plaques and improving cognitive function in Alzheimer's patients, renewing interest in Aβ immunotherapy as a therapeutic approach for the disease despite many previous studies failing to show significant improvements, and the FDA has approved both drugs for treatment [155, 156].

#### 7.1.1 Active anti-Aβ immunotherapies

Vaccination has been shown to have longer-lasting effects than passive immunotherapy using antibodies. Lecanemab is a promising antibody that selectively binds to larger soluble Aβ protofibrils, depleting biotoxic substances and clearing deposited plaques in AD pathology [156-158]. However, clinical trials have reported adverse reactions, including brain hemorrhage and swelling. AV-1959D and DNA vaccines targeting Aβ have shown effectiveness in animal models, but challenges in producing high-titer protective antibodies and evoking a long-lasting antibody response remain [159]. Unresolved issues in the clinical development of an anti-Aβ vaccine include [160] (1) the difficulty of producing a high titer of protective antibodies, (2) the unknown threshold levels of anti-Aβ antibodies, and (3) the difficulty of evoking a long-lasting antibody response in all participants. The complexity of AD, interindividual variability, and a lack of in-depth understanding of its pathophysiology make it a difficult disease to address effectively.

#### 7.1.2 Passive anti-Aβ immunotherapies

The most promising approach for treating AD remains passive immunotherapy strategy, which targets oligomeric conformations. Antibodies entering the brain activate microglia to phagocytose Aβ [9], inhibit Aβ aggregation and promote the depolymerization of Aβ fibers [161]. Antibodies in the blood bind Aβ and reduce the level of free Aβ in the blood. Thus, antibodies can alter the balance of free Aβ on both sides of the blood–brain barrier and enhance the outflow of Aβ from the brain[9]. However, introducing antibodies into the brain can have negative effects, including inflammation and vascular wall damage in the CNS caused by the formation of antigen-antibody complexes in the brain. This "dust-raising effect" occurs when antibodies promote the depolymerization of Aβ fibers and antibodies cross-bind to APP on neuronal membranes, which permits Aβ production and immune attack on neurons [162]. It is suspected that Aβ-specific antibodies exert their effects by attaching them to extracellular oligomers that limit the progression of the illness but do not bind to plaques. Therefore, antibodies with specific affinity for soluble Aβ oligomers may be more successful treatment agents than antibodies with high affinity for monomeric Aβ, fibrillar Aβ, or both forms [161]. This strategy is intriguing because it can sidestep several issues with active immunization, such as T-cell-mediated cytotoxicity or interindividual variations in antibody titer or antibody specificity. Moreover, antibodies are believed to exert anti-inflammatory effects by inhibiting the synthesis of proinflammatory cytokines [153]. These antibodies do not need to enter cells to inhibit or eradicate the transmission of Aβ oligomers from cell to cell by preventing the oligomers from initiating inflammation or spreading disease to other locations [163]. In the future, immunotherapy that mimics hazardous oligomeric Aβ seed epitopes may provide effective protection against amyloid peptides.

### 7.2 Gene therapy

APP mutations can directly cause AD-related abnormalities [164]. Targeted eradication or repair of genetic abnormalities with CRISPR–Cas9 holds potential as a precise and disease-modifying technique for FAD [165]. In animal models of AD, the CRISPR–Cas9 genome editing technique decreases amyloid-associated neurodegeneration [166]. The innovative CRISPR/Cas9 technology can provide effective APP mutation-targeted brain repair via AAV-mediated gene editing, ameliorating the clinical symptoms of AD neurodegeneration. Gene therapy methods for AD have reached phase I/II clinical trials using AAV vectors, which can drive long-term gene expression and enhance synaptic function and structure in rodent models with preexisting amyloidosis[167]. Sustained AAV-mediated overexpression of sAPPα in the brains of elderly APP/PS1dE9 mice with preexisting amyloidosis enhances synaptic function and structure [168] and reverses behavioral impairments [169], suggesting that sAPPα can have therapeutic benefits in rodent models even after the beginning of disease [170]. However, the efficacy of gene editing technology needs improvement to better target amyloid plaques, which are believed to initiate AD neurodegeneration.

### 7.3 Targeting secretases responsible for APP cleavage

#### 7.3.1 Activation of α-secretase

Activating α-secretase reduces APP processing via the amyloidogenic pathway and increases soluble APP, which has neuroprotective and synaptogenesis-promoting properties. Medications such as cholinesterase inhibitors [171], etazolate [172], and non-steroidal anti-inflammatory drugs (NSAIDs) [173, 174] can increase sAPPα and decrease Aβ levels. While ADAM10 activation as a therapeutic strategy for Alzheimer's disease by cleaving APP shows potential, careful evaluation of the potential effects on other cellular processes, such as impaired cell–cell interactions and altered signaling pathways, is crucial to weigh the benefits and risks of targeting ADAM10 activity [175]. However, the effectiveness of α-secretase activation as an anti-Aβ treatment method remains inconclusive and discouraging.

#### 7.3.2 β-secretase inhibitors

In the amyloidogenic pathway, BACE1 initiates APP processing to generate Aβ. BACE1 inhibition is a proven therapeutic target for reducing Aβ production in early AD [176]. However, BACE1 has many other substrates, making the development of targeted treatments challenging. The bulk of initial BACE1 inhibitors were problematic due to low bioavailability and poor penetration across the blood–brain barrier. Second-generation BACE1 inhibitors (verubecestat [177], lanabecestat [178], atabecestat [179], LY3202626 [180], umibecestat [181]and elenbecestat [182]) were developed to be more lipophilic, and several have entered late-stage clinical trials but failed to show any cognitive or functional benefit in individuals with early AD or mild-to-moderate AD. These inhibitors decrease plasma and CSF Aβ levels and brain plaques but do not demonstrate clinical benefits.

#### 7.3.3 Inhibitors and modulators of γ-secretase

γ-Secretase is a potential therapeutic target responsible for the final processing of APP along the amyloidogenic pathway [183]. Inhibitors [184], such as BMS-299897 [185], LY-411575 [186], and LY-450139 [187], have been found to decrease Aβ production. However, targeting γ-secretase must consider avoiding interference with the Notch signaling pathway, which is also digested by the enzyme. JLK isocoumarins [188] and certain NSAIDs have shown promise in reducing Aβ levels without impacting Notch signaling. Future medications should have sufficient potency, brain penetration, or selectivity to reduce brain Aβ levels while avoiding Notch-related toxicity.

#### 7.3.4 Inhibition of Aβ aggregation promotes Aβ degradation and transport

Aβ aggregation plays a significant role in the onset and course of AD, making inhibition of this process a vital therapeutic strategy [189]. Various medications have been demonstrated to decrease Aβ aggregation, such as β-cyclodextrin [190], rifampicin [191] and its derivatives, 4-aminophenol [192], and high levels of taurine [193]. Nicotine and sheet-breaker peptides are also effective [194]. Dual-action small molecules can prevent Aβ aggregation by binding to the β-sheet and sequestering naturally disordered Aβ [195] Screening for nontoxic small molecule drugs for long-term administration may be useful for discovering drugs to prevent or delay the onset of AD. Additionally, several proteases, such as neprilysin [196], insulin-degrading enzyme [197], plasmin [198], endothelin-converting enzyme [199], angiotensin-converting enzyme [200], cathepsin D [201], and metalloproteinase 9 [202], can dissolve amyloid aggregates and plaques.

#### 7.3.5 Inhibition of APP endocytosis reduces Aβ production

APP N-terminal blockers have been shown to effectively inhibit the interaction with ligand proteins that bind to the APP-N-terminus, reduce the metabolic synthesis of Aβ following APP endocytosis, and prevent the development and progression of AD. For instance, our preliminary research has shown that APP operates as a key receptor for the ApoE protein and enhances its endocytosis. The APP-N-terminus-blocking peptide 6KApoE effectively inhibits the entry of ApoE into cells [59]. Inhibiting the binding of the N-terminal portion of the APP protein to its ligands and decreasing APP endocytosis to generate Aβ will be a crucial therapeutic strategy for AD. Several compounds have been identified that target APP N-terminal and influence APP processing to reduce Aβ levels. Donepezil , a cholinesterase inhibitor, has been shown to increase SNX33 expression [203] resulting in elevated levels of sAPPα and surface sAPPα, but not overall APP concentrations, and significantly reduce Aβ levels in cell culture media [204]. Justicidin A decreases APP endocytosis, leading to an increase in APP concentrations and a decrease in Aβ concentrations [205]. Lovastatin inhibits APP endocytosis, most likely through its pleiotropic effects on endocytic regulators, hence decreasing APP β-cleavage and Aβ production [206]. Caffeine also provides some protection against the amyloidogenic processing of APP by blocking the A3R-mediated internalization of APP [207]. Therefore, further exploration and development of APP N-terminal blockers could lead to new therapeutic strategies for AD treatment.

## 8. Future perspectives and conclusions

APP is a transmembrane protein that is well-known as a precursor of Aβ peptides, which accumulate in the brains of AD patients [208]. Since the amyloid cascade hypothesis for AD was established, Aβ has been widely explored. However, in addition to Aβ, APP and its proteolytic cleavage products have different clinical and physiological activities, according to prior research [209]. AD is a complex disease that progresses rather slowly, but the majority of people are diagnosed too late for effective treatment [210]. Therefore, AD pathology must be approached from multiple perspectives, including amyloid and tau pathology and their interactions, inflammatory processes, oxidative stress, insulin resistance, cholesterol, and mitochondrial dysfunction, among other variables [211-213]. Given that these factors are intertwined in multiple ways throughout the life of a patient with AD, early intervention is critical, and multitargeted treatments should be tested in conjunction with the use of conformational antibodies, active immunization, and innovative biomarkers and neuroimaging techniques.

It is now evident that APP and its fragments play a variety of roles in development, cell growth, cell adhesion, intercellular communication, signal transduction, mitochondrial fission and fusion, nuclear signaling, and structural and functional plasticity and that their dysregulation disrupts healthy cellular function [18, 51, 213, 214]. Therefore, it is not surprising that altered APP processing may influence brain function through a range of altered cellular and molecular mechanisms. APP is also crucial for understanding the pathophysiology of AD due to its biochemical, genetic, and neuropathological connections to the illness [215, 216]. APP is the principal source of Aβ, a substantial component of SPs in AD brains. FAD is caused by mutations in the APP gene, including APP locus duplications, emphasizing the importance of APP gene dosage in AD [217, 218]. In individuals with DS (trisomy of chromosome 21), an extra copy of the APP gene causes widespread AD neuropathology. Our recent research revealed that APP may operate as a membrane receptor for ApoE and contribute to Aβ clearance, as demonstrated by our recent research[59]. APP may also function as a receptor for the absorption of tau monomers or aggregates into cells, hence facilitating seed-dependent intracellular tau aggregation [219]. In conclusion, APP contributes significantly to the onset and progression of AD.

Despite the knowledge gained thus far, numerous issues remain to be addressed in understanding the intracellular and extracellular functions of APP for the treatment of AD. First, it is essential to understand the presynaptic and postsynaptic functions of APP family proteins. requiring the identification of specific interaction partners and a more targeted technique in which APP is selectively removed either at the presynaptic or postsynaptic region. Second, the relationship between APP adhesion activities and ectodomain secretion needs to be understood. Third, sophisticated functional studies are necessary to assess the composition and relevance of APP-containing signaling complexes, which may vary among cell types and brain regions. Fourth, we must assess the likelihood that AD pharmacotherapies that target APP processing affect the endogenous functions of APP family members. Finally, additional experimental studies, including those utilizing various methods of viral vector administration, dosage optimization, and tests with larger animal models, are necessary to determine the therapeutic potential of APP for damaged AD neurons. In conclusion, APP plays a significant role in transporting and clearing pathogenic proteins linked with AD, and experiments altering its functions will provide vital insights for the development of AD treatments.

## Supplementary Materials

The Supplementary data can be found online at: www.aginganddisease.org/EN/10.14336/AD.2023.0308.


