# Supplementary material for: Amyloid Precursor Protein: A Regulatory Hub in Alzheimer's Disease
Source: Aging Dis. 2024 Feb 1;15(1):201–25. doi: 10.14336/AD.2023.0308 (PMC10796103; doi:10.14336/AD.2023.0308)
Supplement: Supplementary file 1 [file AD-15-1-201-s.pdf]

SUPPLEMENTARY DATA

Amyloid Precursor Protein: A Regulatory Hub in Alzheimer's Disease

Jiang Chen, Jun-Sheng Chen, Song Li, Fengning Zhang, Jie Deng, Ling-Hui Zeng, Jun Tan

© 2023. Chen J et al. Published online at http://www.aginganddisease.org/EN/10.14336/AD.2023.0308

SUPPLEMENTARY DATA

Supplementary Table 1. The three-dimension structural domains of amyloid precursor protein and their functions.

Domain	Structural Features	Functional Characteristics	References
Extracellular Domain	Copper-binding domain: small β-sheet and helix; Heparin-binding domain: β-sheet and flexible loop; Growth factor-like domain: β-sheet and short α-helix	Regulates APP function and interaction with other proteins and extracellular matrix components	[241], [242], [243]
Transmembrane Domain	Single-pass α-helix spanning the cell membrane; amphipathic, with hydrophobic residues facing the membrane and hydrophilic residues facing the cytoplasm and extracellular environment	Important for stability and localization of APP within the cell membrane and for interaction with other transmembrane proteins	[244], [245]
Juxtamembrane Domain	Located immediately adjacent to the transmembrane domain; contains a GxxxG motif that promotes dimerization of APP and interaction with other transmembrane proteins	Involved in regulation of APP trafficking and processing	[37],[246]
Cytoplasmic Domain	Short and contains several conserved motifs, including the YENPTY motif	Regulates APP trafficking and processing and interacts with intracellular signaling molecules	[247]

© 2023. Chen J et al. Published online at http://www.aginganddisease.org/EN/10.14336/AD.2023.0308
